# *Acrocomia aculeata* associated with doxorubicin: cardioprotection and anticancer activity

**DOI:** 10.3389/fphar.2023.1223933

**Published:** 2023-08-16

**Authors:** Tamaeh Monteiro-Alfredo, Jéssica Maurino dos Santos, Kátia Ávila Antunes, Janielle Cunha, Debora da Silva Baldivia, Ana Salomé Pires, Inês Marques, Ana Margarida Abrantes, Maria Filomena Botelho, Lúcia Monteiro, Ana Cristina Gonçalves, Wellington Henrique Botelho, Ana Paula de Araújo Boleti, Célia Cabral, Paulo J. Oliveira, Edson Lucas dos Santos, Paulo Matafome, Kely de Picoli Souza

**Affiliations:** ^1^ Research Group on Biotechnology and Bioprospection Applied to Metabolism and Cancer (GEBBAM), Federal University of Grande Dourados, Dourados, Brazil; ^2^ Faculty of Medicine, Institute of Physiology, University of Coimbra, Coimbra, Portugal; ^3^ Faculty of Medicine, Coimbra Institute for Clinical and Biomedical Research (iCBR), University of Coimbra, Coimbra, Portugal; ^4^ Clinical Academic Center of Coimbra (CACC), University of Coimbra, Coimbra, Portugal; ^5^ Center for Innovative Biomedicine and Biotechnology (CIBB), University Coimbra, Coimbra, Portugal; ^6^ Faculty of Medicine, Coimbra Institute for Clinical and Biomedical Research (iCBR) Area of Environment Genetics and Oncobiology (CIMAGO), Institute of Biophysics, University Coimbra, Coimbra, Portugal; ^7^ Faculty of Pharmacy, University of Coimbra, Coimbra, Portugal; ^8^ CNC—Center for Neuroscience and Cell Biology, Center for Innovative Biomedicine and Biotechnology, University of Coimbra, Coimbra, Portugal; ^9^ Department of Complementary Sciences, Instituto Politécnico de Coimbra, Coimbra Health School (ESTeSC), Coimbra, Portugal

**Keywords:** antioxidant, oxidative stress, chemotherapy side-effects, bocaiúva, macaúba, Brazilian cerrado

## Abstract

Doxorubicin (Dox) is a chemotherapeutic agent widely used in the clinic, whose side effects include cardiotoxicity, associated with decreased antioxidant defenses and increased oxidative stress. The association of Dox with natural antioxidants can extend its use if not interfering with its pharmacological potential. In this study, we aimed to understand the effects and mechanisms of the aqueous extract of *Acrocomia aculeata* leaves (EA-Aa) in cancer cells and the co-treatment with Dox, in *in vitro* and *in vivo* models. It was found that EA-Aa showed a relevant decrease in the viability of cancer cells (K562 and MCF-7) and increased apoptosis and death. The Dox cytotoxic effect in co-treatment with EA-Aa was increased in cancer cells. The therapeutic association also promoted a change in cell death, leading to a higher rate of apoptosis compared to the Dox group, which induced necrosis. In addition, in non-cancer cells, EA-Aa enhanced red blood cell (RBC) redox state with lower hemolysis and malondialdehyde (MDA) content and had no *in vitro* nor *in vivo* toxicity. Furthermore, EA-Aa showed antioxidant protection against Dox-induced cytotoxicity in H9c2 cells (cardiomyoblast), partially mediated by the NRF2 pathway. *In vivo*, EA-Aa treatment showed a relevant decrease in MDA levels in the heart, kidney, and brain, evaluated in C57Bl/6 mice induced to cardiotoxicity by Dox. Together, our results proved the effectiveness of EA-Aa in potentiating Dox anticancer effects, with antioxidant and cardioprotective activity, suggesting EA-Aa as a potential Dox pharmacological adjuvant.

## 1 Introduction

Doxorubicin (Dox), a potent chemotherapeutic anthracycline (Cai et al., 2020), was first extracted from *Streptomyces peuceutius* var. *caesius* in the mid-60s (Benjanuwattra et al., 2020), and nowadays it is used in the treatment of various types of cancer, such as breast, lung (Cai et al., 2020), gastric, ovarian, pancreatic cancer and hematologic malignancies (Benjanuwattra et al., 2020). Among the mechanisms for Dox anticancer effects, the production of reactive oxygen/nitrogen species (ROS/RNS), apoptosis induction due to cytochrome C release, and DNA double-strand breaks have been identified (Cai et al., 2020). These effects can also partly explain the cytotoxicity of the drug in non-target tissues, namely, the heart. Cardiotoxicity is a recognized side effect associated with Dox therapy (Cai et al., 2020), that reduces patient quality of life and adherence to the treatment (Shabalala et al., 2017). Besides the increased ROS/RNS production, Dox cardiotoxicity is linked to decreased cardiac antioxidant defense (Alanazi et al., 2020), which leads to a pro-oxidative condition and mitochondrial dysfunction (Wallace et al., 2020).

Several studies carried out with medicinal plants that have a proven antioxidant effect have shown that the co-administration of plant extracts and doxorubicin can reduce its cardiotoxicity (Wattanapitayakul et al., 2005; Yu et al., 2018; Cheng et al., 2022). The presence of phenolic compounds in these plant extracts and the activation of the nuclear factor erythroid 2-related factor 2 (NRF2) have been described as responsible for this protective effect (Lin et al., 2019; Yarmohammadi et al., 2021). The NRF2 is a transcription factor that regulates several signaling pathways associated with oxidative stress, such as the expression of antioxidant genes and phase II detoxifying enzymes (Sharma et al., 2020). There are reports that the reduction of NRF2 levels is associated with more significant Dox-induced cardiotoxicity, in addition to increased cardiac dysfunction (Li et al., 2014). Consequently, compounds that increase NRF2 levels and activity generate excellent protection against Dox cardiotoxicity (Tomlinson et al., 2019), making this transcriptional factor an important target in the treatment of Dox-induced cardiotoxicity (Han et al., 2008; Li et al., 2015; Bai et al., 2016). Among NRF2 activators, phenolic compounds are a strategic therapeutic target that can help improve the adverse conditions associated with Dox chemotherapy (Han et al., 2008).

*Acrocomia aculeata* Jacq. (Lodd) ex Mart., a palm native from Brazil, commonly known as macaúba or bocaiúva, has several therapeutic uses described in the literature for its pulp fruit, and almond (Agostini-Costa, 2018). Recently, our group demonstrated the hypoglycemic (Monteiro-Alfredo et al., 2021) and antioxidant potential of the aqueous extract of its leaves (EA-Aa). In addition to demonstrating its therapeutic properties, we also describe its chemical composition, highlighting the majority presence of phenolic compounds and flavonoids (Monteiro-Alfredo et al., 2020), namely quercetin (Li et al., 2018), vanillic (Baniahmad et al., 2020), ferulic and caffeic acid (Chegaev et al., 2013). These compounds have been separately associated with protective effects against Dox-induced toxicity (Razavi-Azarkhiavi et al., 2016; Benzer et al., 2018; Yousefian et al., 2022). In this context, we aimed to evaluate the effects and mechanisms of EA-Aa in cancer cells by itself and in the co-treatment with Dox, *in vitro* and *in vivo* models.

## 2 Materials and methods

### 2.1 Reagents and chemicals

The organic solvents and salts used in experiments were purchased from Merck/Sigma-Aldrich, Biowest, Gibco, Lonza, and Fischer Scientific.

### 2.2 Plant material and extract preparation

*A. aculeata* fresh leaves were collected as mentioned before (Monteiro-Alfredo et al., 2020) in the region of Grande Dourados, Macaúba district, state of Mato Grosso do Sul (MS) (22°0702.4 S 54°2836.3 W), under the permission of the Brazilian Biodiversity Authorization and Information System (Sistema de Autorização e Informação sobre Biodiversidade, SISBIO; no. 50589). The identification of the species was performed by a plant taxonomist, followed by the placing of a voucher specimen in the herbarium (DDMS-UFGD) of the Federal University of Grande Dourados, Dourados (MS), Brazil, registration number—5103. The aqueous extract was prepared as previously described (Monteiro-Alfredo et al., 2020). In summary, for the preparation of the aqueous extract of *A. aculeata* (EA-Aa), leaves were collected, washed, dried, crushed, and the extract was prepared by infusion (100 g.L^−1^), freeze-dried and stored at −20°C.

### 2.3 Cell culture

Human chronic myeloid leukemia (K562) and breast cancer (MCF-7), cultivated in Roswell Park Memorial Institute Medium (RPMI-1640, Sigma, United States) with 10% fetal bovine serum (FBS) and Dulbecco’s Modified Eagle’s Medium (DMEM, Sigma, United States) 5% FBS, respectively, were used. Normal cells from rat cardiomyoblasts (H9c2) were cultured with DMEM and FBS 10%. Human peripheral mononuclear cells (PBMC) were isolated from a healthy adult donor. For the isolation of PBMC, peripheral blood was collected and mixed with Ficoll-Paque at a 1:1 ratio and centrifuged at 800 × g, 30 min. The PBMC layer was collected and washed twice with phosphate saline buffer (PBS), centrifuged at 330 × g, 10 min, and the cell pellet was resuspended and cultivated in RMPI 20%. All culture media were supplemented with 1% penicillin/streptomycin and cells cultivated at 37°C and 5% CO_2_.

#### 2.3.1 Assessment of cell viability

Cell viability was determined through cellular metabolic activity using the Alamar Blue assay. K562 cells (2 × 10^4^ cells.well^−1^), MCF-7 cells (1 × 10^4^ cells.well^−1^), H9c2 cells (3 × 10^4^ cells.well^−1^) and PBMC cells (12 × 10^4^ cells.well^−1^) were seeded in 96-well plates, and treated with EA-Aa (31.25–500 μg mL^−1^ diluted in the respective cell culture medium) with or without Dox (IC_20_ = 0.5 μg mL^−1^ and IC_50_ = 1 μg mL^−1^, diluted in the respective cell culture medium) for 24 h or 48 h. After reaching the incubation time, suspension cells (K562 and PBMC) were centrifuged at 2000 rpm, 20 min, and the medium was replaced by a solution of RPMI 10% or 20% with 10% of resazurin (0.1 mg mL^−1^); Adherent cells (MCF-7 and H9c2) had the medium replaced by the same solution of resazurin in DMEM 5% or 10%. After the reagent conversion period, the absorbance was measured at 570 nm and 600 nm in a BioTek microplate reader (BioTek Instruments, Inc., Winooski, VT, United States).

To determine the role of NRF2 in cardiomyoblast antioxidant protection, the NRF2 inhibitor, ML385 (20 μM, diluted in DMEM 10%), was previously added to H9c2 cells (24 h), followed by EA-Aa (31.25–500 μg mL^−1^) and Dox (IC_50_ = 20 μg mL^−1^) this value was obtained from a dose-response curve of doxorubicin, specifically prepared in H9c2 cells) incubations for 24 h. After this period, the cell viability was determined by Alamar Blue assay. The results obtained by the Gen5 program were used to calculate cell viability, according to Eq. 1 (Monteiro-Alfredo et al., 2020). Three independent experiments were performed in triplicate.

$$Cell\;metabolic\;activity=\left(\frac{\left({Abs}_{570}-{Abs}_{600}\right)of\;treated\;cells}{\left({Abs}_{570}-{Abs}_{600}\right)of\;control\;cells}\right)\times 100$$ (1)

#### 2.3.2 Flow cytometry

The flow cytometry assays were all performed on the leukemia cell line, K562 (1 × 10^6^ cells.well^−1^), analyzed in a four-color FACSCalibur flow cytometer (Becton Dicksson, United States) and Paint-A-Gate Software (Becton Dicksson, United States).

##### 2.3.2.1 Cell death

To determine cell viability and death, K562 cells were treated with EA-Aa (250–500 μg mL^−1^) and Dox (IC_20_ = 0.5 μg mL^−1^ and–IC_50_ = 1 μg mL^−1^) for 48 h. After treatment, to assess the mentioned parameters, cells were incubated with annexin-V (an-V, Immunostep, Spain) and propidium iodide (PI, Immunostep, Spain). Fluorescein isothiocyanate conjugates an-V and PI were used to label cells. Data were expressed in % of viable (an-V-/PI-), apoptotic (an-V+/PI-), late apoptotic/necrotic (an-V+/PI+), and necrotic cells (an-V-/PI+) as previously described (Pires et al., 2016).

##### 2.3.2.2 Mitochondrial membrane potential (ΔΨmt)

Mitochondrial membrane potential was assessed with the fluorescent probe 5,5,6,6-tetrachloro-1,1,3,3-tetraethyl benzimidazolocar-bocyanine iodide (JC-1, Sigma, United States). K562 cells treated with EA-Aa (250–500 μg mL^−1^) and Dox (IC_20_ = 0.5 µg.mL^−1^ and–IC_50_ = 1 μg mL^−1^) for 48 h were incubated with JC-1 for 15 min, in the dark, at 37°C, before flow cytometry evaluation (Pires et al., 2016). Data are presented as the ratio of aggregates/monomers (A/M), which is proportional to the mitochondrial membrane potential, as previously described (Pires et al., 2016).

#### 2.3.3 Intracellular ROS measurement

To determine the effect of EA-Aa on ROS formation in cardiomyoblasts, H9c2 cells (3 × 10^4^ cells.well^−1^) were seeded in MilliCells^®^ EZ Slide 8-well glass (Millipore, United States). After reaching 80% of confluence, cells were treated with EA-Aa (125, 250, and 500 μg mL^−1^) for 30 min, followed by the addition of Dox (IC_20_ = 0.5 μg mL^−1^ and IC_50_ = 1 μg mL^−1^) overnight. The evaluation of intracellular ROS was carried out with 2,7-dichlorodihydrofluorescein diacetate (H_2_DCFDA, Invitrogen, United States), following the manufacturer’s instructions. DAPI was used to stain the cell nucleus. Images were obtained with a fluorescence microscope (Zeiss Axio Observer Z1) with an incorporated camera (Zeiss, Germany), detected with 504 nm of excitation and 525 nm of emission for DCF and 353 nm of excitation, and 465 nm of emission for DAPI (Sigma, United States). The settings were the same for all analyses. The quantification was performed in the entire image with the software ImageJ.

### 2.4 Oxidative hemolysis assay

#### 2.4.1 Dox-induced *in vitro* oxidative hemolysis assay

After the approval of the Research Ethics Committee CEP/UFGD no 5160, peripheral blood was collected from a single adult healthy donor and stored in tubes containing the anticoagulant sodium citrate. A solution of 10% of red blood cells (RBC) in physiological solution (NaCl 0.9%) was prepared and previously incubated with EA-Aa in different concentrations (31.25–500 μg mL^−1^) at 37°C for 30 min, under constant shaking. Subsequently, the RBC solution was incubated with Dox (Sigma, United States—300 μg mL^−1^ diluted in 0.9% NaCl—concentration determined by the IC_50_ obtained in a previous Dox hemolysis assay) for 4 h. Dox was used as an inductor of oxidative stress. After centrifugation (3,000 rpm), the supernatant was read at 540 nm, and the results were expressed as a percentage of hemolysis based on total hemolysis (incubation of RBC and distilled water). Three independent experiments were performed in duplicate as previously described (Monteiro-Alfredo et al., 2020).

#### 2.4.2 Evaluation of Dox-induced malondialdehyde (MDA) generation *in vitro*

Following Dox-induced oxidative hemolysis, as previously described, an aliquot of the supernatant of the same sample was mixed with 20 nM of thiobarbituric acid (TBA, Merck, Germany), incubated at 96°C for 45 min and then placed in an ice bath for 15 min to stop the reaction. Butanol was added to the tubes to extract the organic fraction of the samples. The absorbance of the supernatant was determined by spectrophotometry at 532 nm. The lipid peroxidation product, MDA, was calculated as Eq. 2, as previously described (Monteiro-Alfredo et al., 2020).

$$MDA\;nmol.{mL}^{-1}=\frac{{Abs}_{sample}\;\left(20\times 220.32\right)}{{Abs}_{standard\;MDA}}$$ (2)

### 2.5 Animals

#### 2.5.1 Animal maintenance

After the approval of the Federal University of Grande Dourados (UFGD) Ethics Committee on Animal Use n^o^ 10/2017, the experiments were conducted following the ethical principles of animal experimentation adopted by the National Council for the Control of Animal Experimentation (Conselho Nacional de Controle de Experimentação Animal, CONCEA). C57Bl/6 mice were maintained under controlled conditions, namely, the temperature of 22°C ± 2°C, 12 h light-dark cycle, and *ad libitum* fed.

#### 2.5.2 EA-Aa acute toxicity determination in C57Bl/6 mice

For the evaluation of the acute toxicity of EA-Aa, the tests were based on protocols from the Organization for Economic Cooperation and Development (OECD) Guideline 425 (Test No. 425). Animals were fasted for 8 h, followed by the administration of one single gavage of EA-Aa (2000 mg kg^−1^) in a female C57Bl/6 mouse. The animal was frequently observed during the first 24 h. As the first animal did not show any symptoms of toxicity, the test was carried out with the administration of the remaining four mice. The same protocol was repeated with a dose of 5000 mg kg^−1^ to define the lethal dose. The control group received only water through gavage (*n* = 5). After administering the treatment, the animals were observed for 14 days, and during this period, body mass, food, and water intake were measured regularly (according to the study design in Figure 6). The Hippocratic screening (dos Santos et al., 2018) was performed to determine behavioral and physiological parameters: urination, defecation, exophthalmos, tremor, catatonia, piloerection, tail erection, hypersalivation, ataxia, lacrimation, pallor/hyperemia/cyanosis of the ears, nose scratching, tail biting, and paw licking. After the study, the animals were anesthetized with ketamine/xylazine (1:1—both in a dose of 100 μL.100 g^−1^) and euthanized by cervical dislocation followed by organ collection (brain, heart, lungs, liver, spleen, and kidneys), weighing, and macroscopical analysis. Blood was also collected for hematological analysis.

#### 2.5.3 Cardiotoxicity induced by Dox in C57/Bl6 mice

To induce *in vivo* cardiotoxicity with Dox, male C57Bl/6 mice (*n* = 5) with weight around 25 g were randomly distributed between 3 groups: 1—Control (water, p.o.); 2—Dox (water p. o.); 3—Dox + EA-Aa (EA-Aa 200 mg.kg^−1^ p.o.). EA-Aa was daily co-administered with Dox (in a cumulative dose of 24 mg kg^−1^ diluted in 0.9% NaCl), which occurred from the 7th day onwards and on alternate days (according to the protocol presented in Figure 7). The animals were euthanized on the 18th day, with the same protocol performed in the acute toxicity test as previously described (dos Santos et al., 2018).

#### 2.5.4 Dosage of MDA levels in C57Bl/6 mice organs

The liver, heart, kidney, and brain were homogenized in 1.15% potassium chloride (KCl) and centrifuged at 3,000 rpm, for 10 min. The supernatant was collected (0.5 mL) and incubated with 1 mL of 10% trichloroacetic acid (TCA) and 1 mL of 20 nM TBA (diluted in 75 nM PBS) at 96°C/45 min. After sample cooling, 3 mL of butanol was added to the tubes, and the mixture was homogenized, centrifuged (3,000 rpm, 5 min), and the absorbance was read (dos Santos et al., 2018).

### 2.6 Statistical analysis

Results were expressed as mean ± standard error of the mean (SEM). All data were compared by One way-ANOVA followed by Student-Newman-Keuls posttest, to compare all means. To evaluate the interactions between cells treated with EA-Aa and Dox, we performed Two way-ANOVA followed by Sidak’s posttest. All results were performed with the software GraphPad Prism 7.0. The data were considered significant when *p* < 0.05.

## 3 Results

### 3.1 EA-Aa reduces cancer cell viability, increases Dox-induced cytotoxicity and mitochondrial membrane potential

**FIGURE 1:**
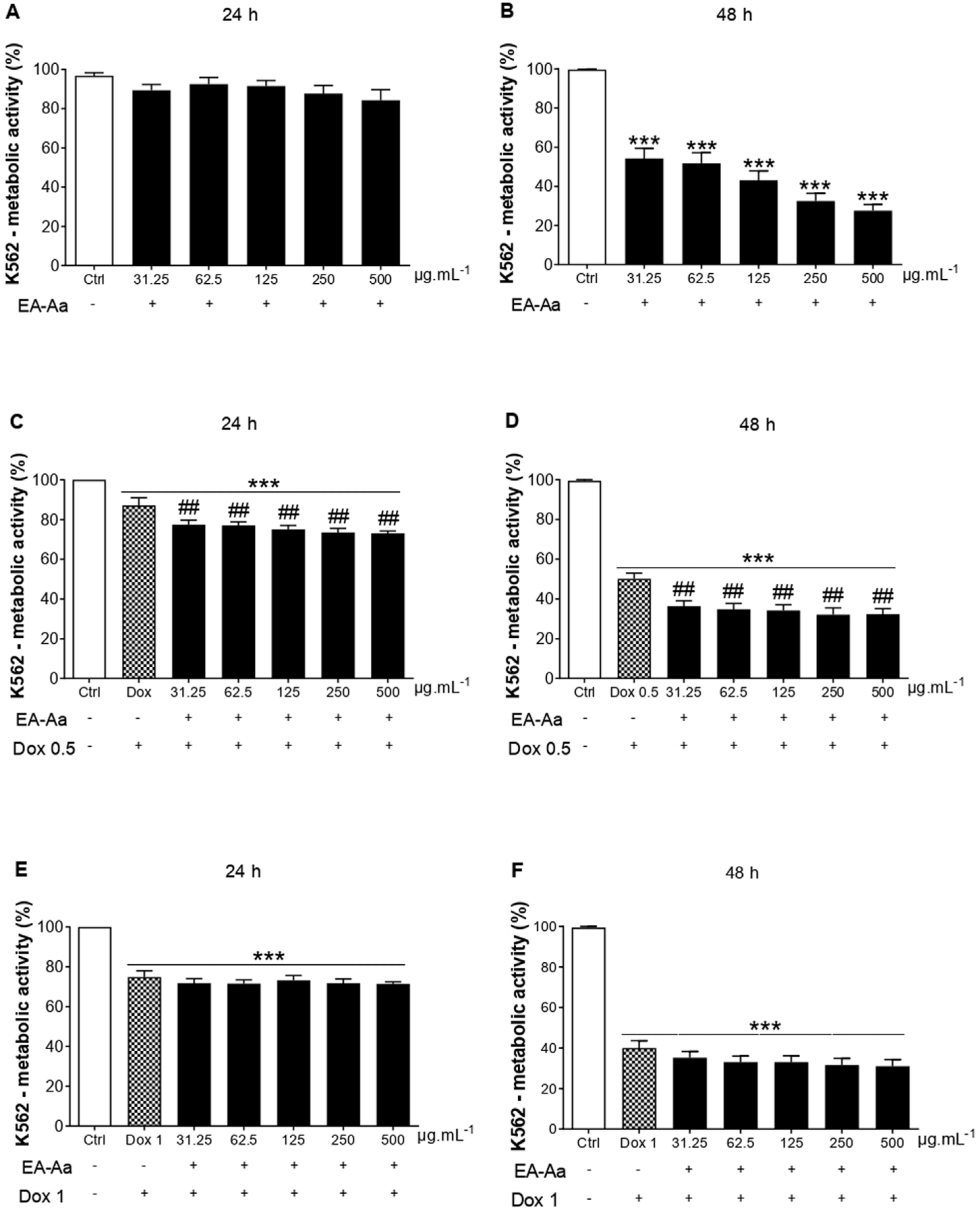
EA-Aa reduces cancer cell survival and proliferation and increases Dox-induced cytotoxicity in Human chronic myeloid leukemia K562 cells. Metabolic activity of EA-Aa-treated (31.25–500 μg mL^−1^) K562 cells determined by the resazurin reduction: 24 h **(A)** and 48 h **(B)**. Co-incubation of EA-Aa with Dox 0.5 μg mL^−1^: 24 h **(C)** and 48 h **(D)**. Co-incubation of EA-Aa with Dox 1 μg mL^−1^: 24 h **(E)** and 48 h **(F)**. * vs. Ctrl; ^#^ vs. Dox 0.5 and 1 μg mL^−1^; ^##^ *p* < 0.01; ***p < 0.001.

**FIGURE 2:**
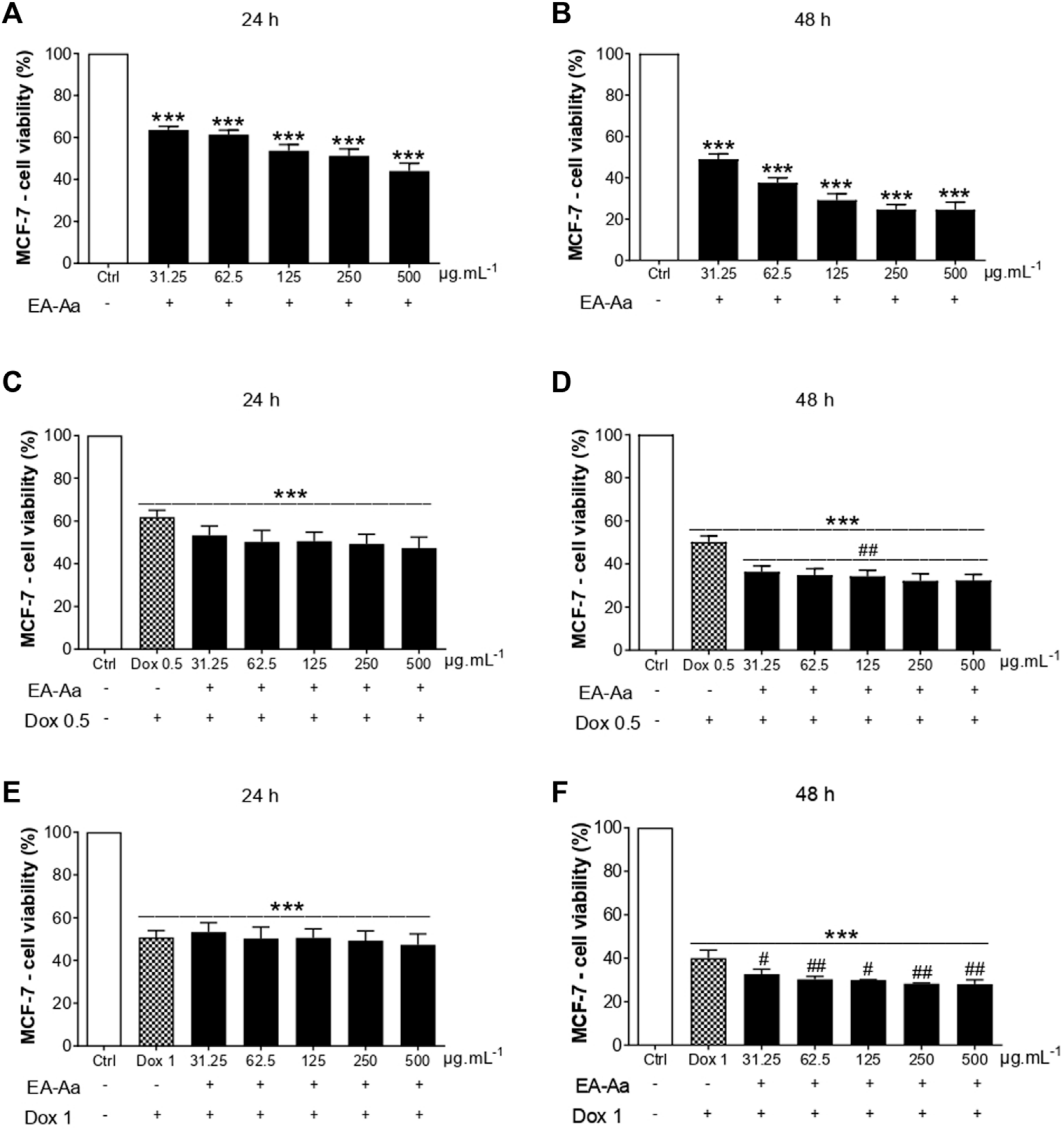
EA-Aa reduces cancer cell survival and increases Dox-cytotoxicity in human breast cancer MCF-7 cells. Metabolic activity of EA-Aa-treated (31.25–500 μg mL^−1^) MCF-7 determined by the resazurin reduction: 24 h **(A)** and 48 h **(B)**. Co-incubation between EA-Aa and Dox 0.5 μg mL^−1^: 24 h **(C)** and 48 h **(D)**. Co-incubation between EA-Aa and Dox 1 μg mL^−1^: 24 h **(E)** and 48 h **(F)**. * vs. Ctrl; ^#^ vs. Dox 0.5 and 1 μg mL^−1^; ^#^ *p* < 0.05; ^##^ *p* < 0.01; ***p < 0.001.

The cytotoxic effect of EA-Aa was observed in K562 and MCF-7 cells. At the highest investigated doses (250 and 500 μg mL^−1^), after 48 h, EA-Aa showed a cytotoxic effect of approximately 73% on K562 cells and approximately 76% on MCF-7 cells, concerning the control (Figure 1B, Figure 2B). In 24 h, EA-Aa reduced the cell viability of MCF-7 between 40% and 50% at the evaluated concentrations but did not affect K562 cells (Figure 1A, Figure 2A). The concentration and time-dependent effects of EA-Aa and Dox observed in K562 and MCF-7 cells show that at concentrations of 0.5 and 1 μg mL^−1^, respectively, there was a reduction in cell viability, compared to the control, by approximately 20%–30% at 24 h and 60% to 48 h in K562 cells (Figures 1C–F) and on average at 50% to 24 h and 60% to 48 h in MCF-7 cells (Figures 2C–F). Considering the action of Dox (0.5 μg mL^−1^) during the investigated times, the cytotoxic effect of Dox reduced cell viability by approximately 10%–50% in K562 cells, and by 40%–50% in MCF-7 cells at the lowest concentration at 24 and 48 h, respectively (Figures 1C, D, Figures 2 C, D). Reductions in cell viability at the highest Dox (1 μg mL^−1^) concentration were approximately 30%–60% in K562 cells and 50%–60% in MCF-7 cells at 24 and 48 h, respectively (Figures 1E, F, Figures 2E, F). In summary, it is observed that doubling the concentration of Dox does not result in a substantial increase in the cytotoxic effect of Dox on cancer cells, neither at 24 nor 48 h of treatment.

The co-treatment of K562 leukemic cells with EA-Aa and the chemotherapeutic agent potentiated the cytotoxicity of Dox, always reducing cell viability at all times and concentrations investigated (Figures 1C, D), except at 24 and 48 h at a concentration of 1 μg mL^−1^ (Figures 1E, F). In MCF-7 cells, only in the 48 h treatment, the effects of the Dox and EA-Aa co-treatment were observed, where there was a reduction in cell viability in the two evaluated Dox concentrations. In short, it is observed that the most potent effects of Dox are observed at the concentration of 1 μg mL^−1^ after 48 h (Figure 2F), with a reduction of approximately 60% in the cell viability of cancer cells, and that the same result, was achieved with half of Dox concentration, associated to EA-Aa, in both cells. It is noteworthy that the best cytotoxicity results were observed with a reduction of approximately 70% in the viability of cancer cells at the highest dose of Dox in co-treatment with EA-Aa and at the longest incubation time (Figure 1F, Figure 2F).

**FIGURE 3:**
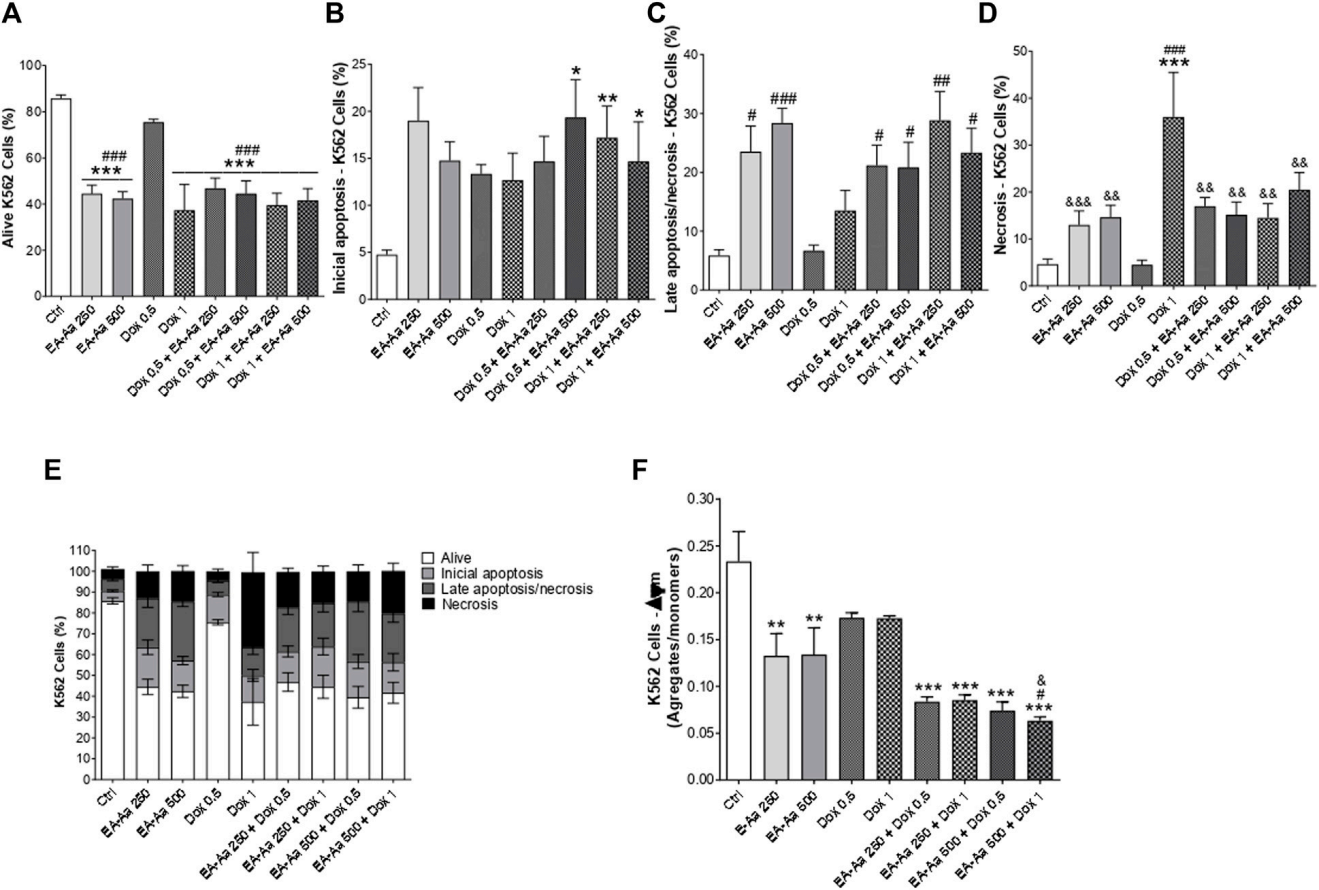
EA-Aa reduces mitochondrial membrane potential and induces apoptosis in K562 cells. Flow cytometry analysis by annexin-V, PI and JC-1 staining. Alive cells **(A)** and types of cell death: Initial apoptosis **(B)**; Late apoptosis/necrosis **(C)**; Necrosis **(D)**; summarized data **(E)**; and JC-1 staining **(F)**. * vs. Ctrl; ^#^ vs. Dox 0.5 μg mL^−1^; ^&^ vs. Dox1 μg.mL^−1^; *, ^#^ *p* < 0.05; **, ^##^, ^&&^ *p* < 0.01; ***, ^###^, ^&&&^ *p* < 0.001.

To evaluate if the decrease in cell survival induced by EA-Aa was associated with an increase in cell death, the effect of EA-Aa (250 and 500  μg mL^−1^) alone and in combination with Dox (0.5 and 1  μg mL^−1^) was analyzed by flow cytometry, in K562 cells using double staining with annexin-V and propidium iodide. Figure 3A shows a lower percentage of alive cells after treatment with EA-Aa (250 and 500 μg mL^−1^). The combination of EA-Aa with Dox (0.5 and 1  μg mL^−1^) decreased cell survival to values like those observed in cells treated with Dox 1 μg mL^−1^ alone (Figure 3A). Data of initial and late apoptosis (Figures 3B, C) showed little effect of Dox, especially in initial apoptosis. This chemotherapeutic agent acted mostly through necrosis (41.8%), especially at 1 μg mL^−1^ (Figure 3D). On the other hand, cells treated with EA-Aa alone presented a large number of cells suffering from apoptosis, while the increase in necrosis was much smaller (Figures 3B–E). The co-incubation of Dox and EA-Aa significantly reduced Dox-induced necrosis, showing 17% and 15.16% in Dox 0.5 + EA-Aa 250/500 μg mL^−1^ and 14.5% and 20.5% in Dox 1 + EA-Aa 250/500 μg mL^−1^, respectively (Figure 3D). Accordingly, co-incubation significantly increased late apoptosis (Figure 3C). These data are summarized in Figure 3E.

Regarding the mitochondrial membrane potential measurements, EA-Aa treatment resulted in a decreased ratio of JC-1 aggregates/monomers, which indirectly assesses that parameter. EA-Aa (in both concentrations) by itself showed a reduction in values compared to the control (Figure 3F). The co-incubation of EA-Aa and Dox presented a more significant reduction in the mitochondrial potential than the cells treated only with Dox or only with EA-Aa, particularly EA-Aa 500 μg mL^−1^, potentiating the effect of Dox 1 μg mL^−1^ (Figure 3F).

### 3.2 EA-Aa prevents Dox-induced oxidative hemolysis and oxidative stress in non-cancer cells

**FIGURE 4:**
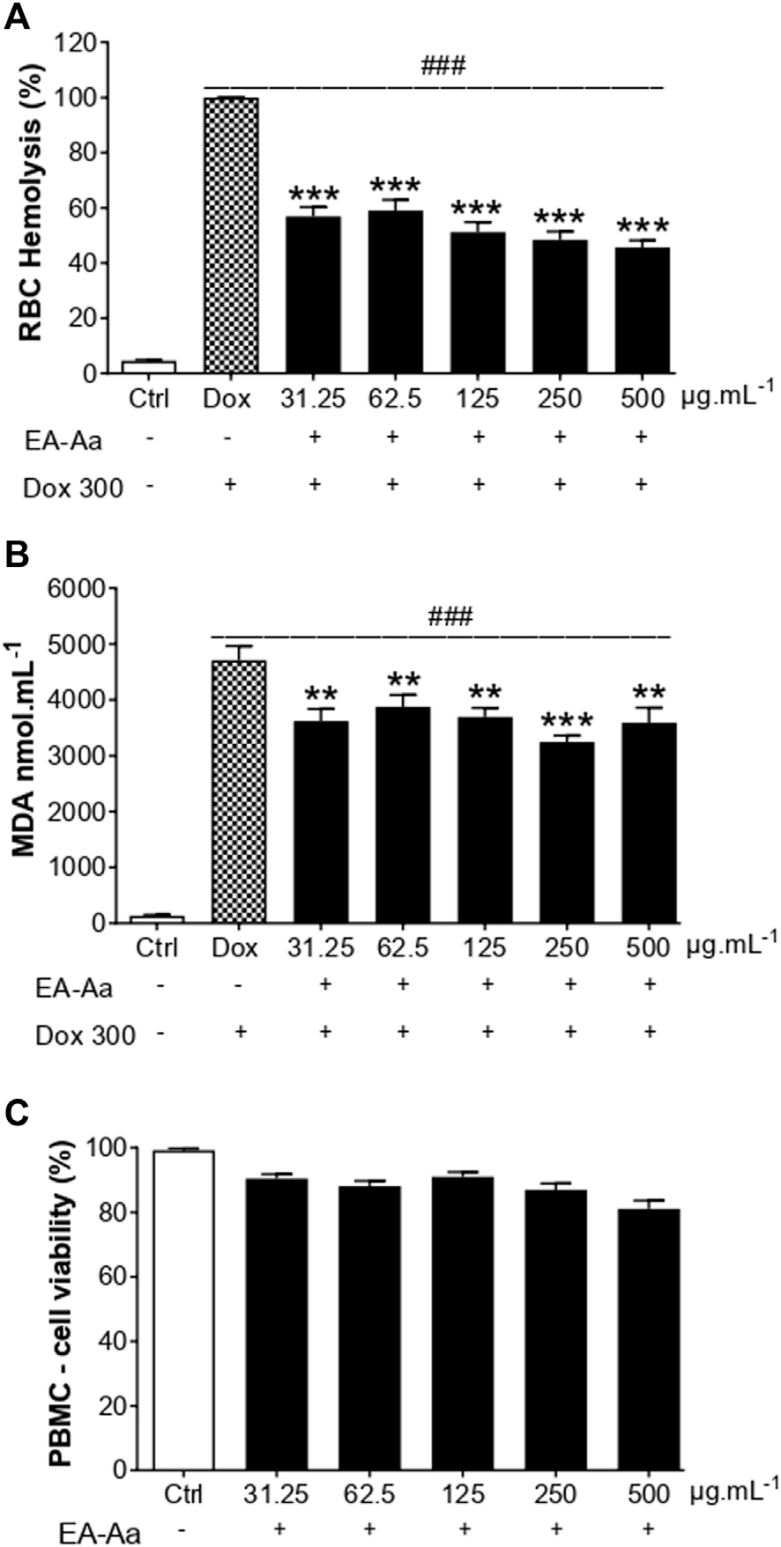
EA-Aa shows no toxicity in blood cells while preventing Doxinduced MDA formation. Hemolysis **(A)** and MDA formation **(B)** in RBCs exposed to Dox 300 μg mL^−1^ and treated with EA-Aa (31.25–500 μg mL^−1^) for 4 h. Metabolic activity of PBMC **(C)** treated with EA-Aa (31.25–500 μg mL^−1^) for 24. * vs. Ctrl; ^#^ vs. Dox. **p < 0.01; ***, ^###^ *p* < 0.001.

After confirming EA-Aa cytotoxic potential in cancer cells, we analyzed its possible toxic effects in normal cells, to exclude potential side effects in non-tumor cells. Dox-induced oxidative hemolysis assay and the generation of MDA in RBC, the protective effects of EA-Aa, and the absence of its toxicity in PBMC are presented in Figure 4. After 240 min of incubation, Dox-induced hemolysis in RBC was significantly reduced by the treatment with EA-Aa, which showed 50% protection compared with Dox-treated cells (Figure 4A). These results are confirmed by the data in Figure 4B, in which EA-Aa decreased by 30% of the Dox-induced MDA levels.

**FIGURE 5:**
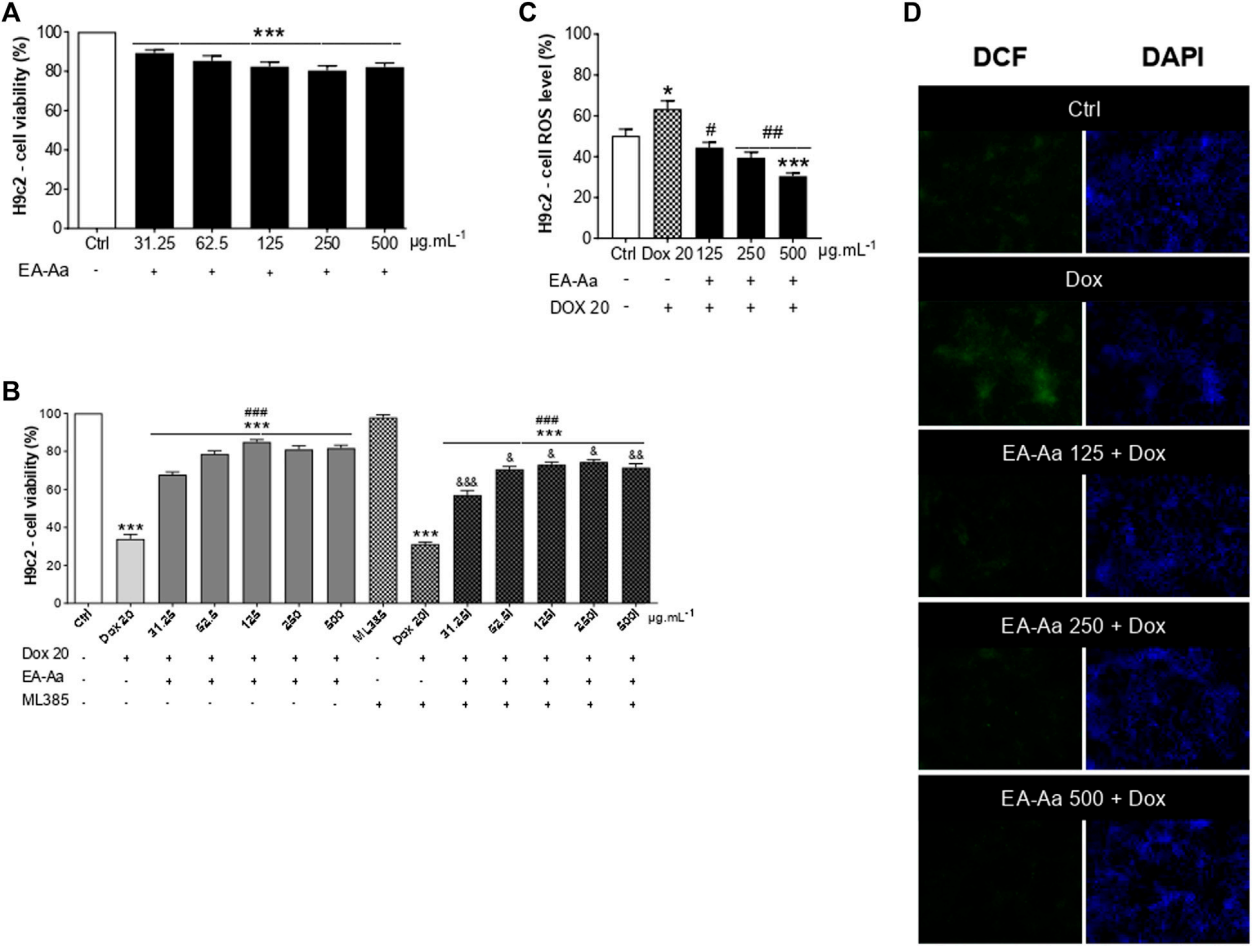
EA-Aa prevents cardiomyoblast H9c2 against Dox-induced oxidative stress and reduces ROS generation. Metabolic activity of H9c2 cells treated with EA-Aa (31.25–500 μg mL^−1^) for 24 h **(A)**. EA-Aa- (31.25–500 μg mL^−1^) and Dox-treated (IC_50_ 20 μg mL^−1^) H9c2 cells, incubated with or without NRF2 inhibitor, ML 385 **(B)**. The intracellular level of ROS in H9c2 cells **(C)** and the respective representative images (DCF, green; DAPI, blue) **(D)**. * vs. Ctrl; ^#^ vs. Dox; ^&^ vs. respective concentration without NRF2 inhibitor, ML 385. *^, #, &^ *p* < 0.05; ^##, &&^ *p* < 0.01; ***^, ###, &&&^ *p* < 0.001.

After the treatment of 24 h, EA-Aa presented a slight decrease in H9c2 cardiomyoblasts viability (Figure 5A), while PBMC did not (Figure 4C). So, we tested the effect of EA-Aa in protecting H9c2 cells from Dox-induced cytotoxicity and oxidative stress (IC_50_ = 20 μg mL^−1^). Data in Figure 5B show that the previous treatment with EA-Aa restored cell viability and prevented the effect induced by the higher dose of Dox (20 μg mL^−1^). The NRF2 pathway partially mediates this condition, since cells incubated with its inhibitor, ML385, had a modest (∼10%) effect in reducing the protective effect of the extract (Figure 5B). Accordingly, when cardiomyoblasts were incubated under the same conditions as cancer cells (Dox 1 μg mL^−1^), they showed prevention of increased ROS levels and further reduction to the basal intracellular levels, as indicated by DCF fluorescence, of about 30%, 38%, and 52% due to EA-Aa treatment (125, 250 and 500 μg mL^−1^, respectively) in comparison with cells treated with Dox alone (Figures 5C, D).

### 3.3 EA-Aa has no acute toxicity and decreases Dox-induced cardiac nephron and neurotoxicity in C57Bl/6 mice

**FIGURE 6:**
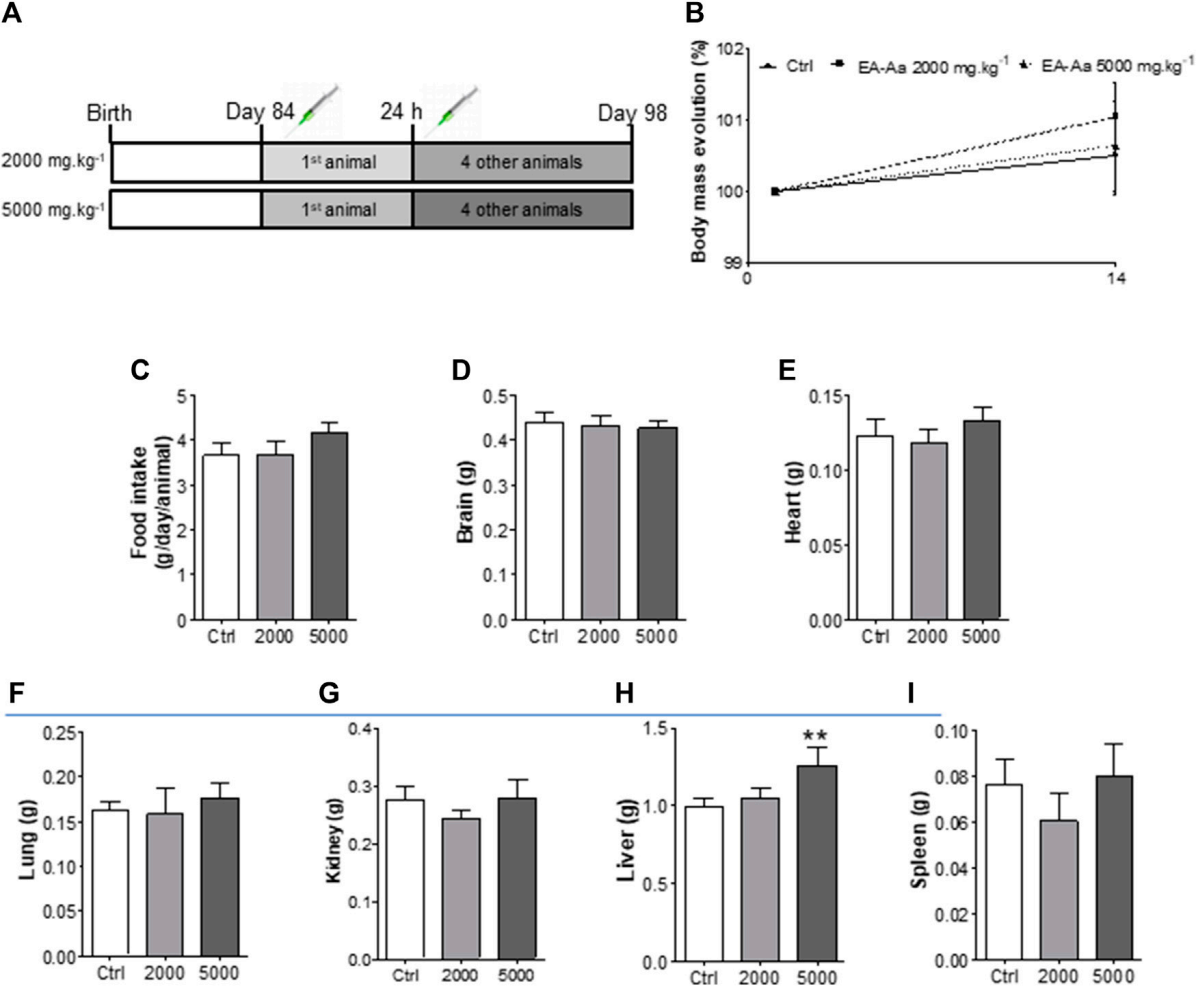
*In vivo* acute toxicity of EA-Aa in C57Bl/6 mice. **(A)** Experimental design; **(B)** Initial and final body mass; **(C)** Food intake; **(D)** Brain, **(E)** Heart, **(F)** Lung, **(G)** Kidney, **(H)** Liver and **(I)** Spleen weights. Ctrl group–control mice; 2000 group–treated mice with EA-Aa 2000 mg.kg^−1^; 5000 group–treated mice with EA-Aa 5000 mg.kg^−1^. * vs. Ctrl; ** *p* < 0.01.

**FIGURE 7:**
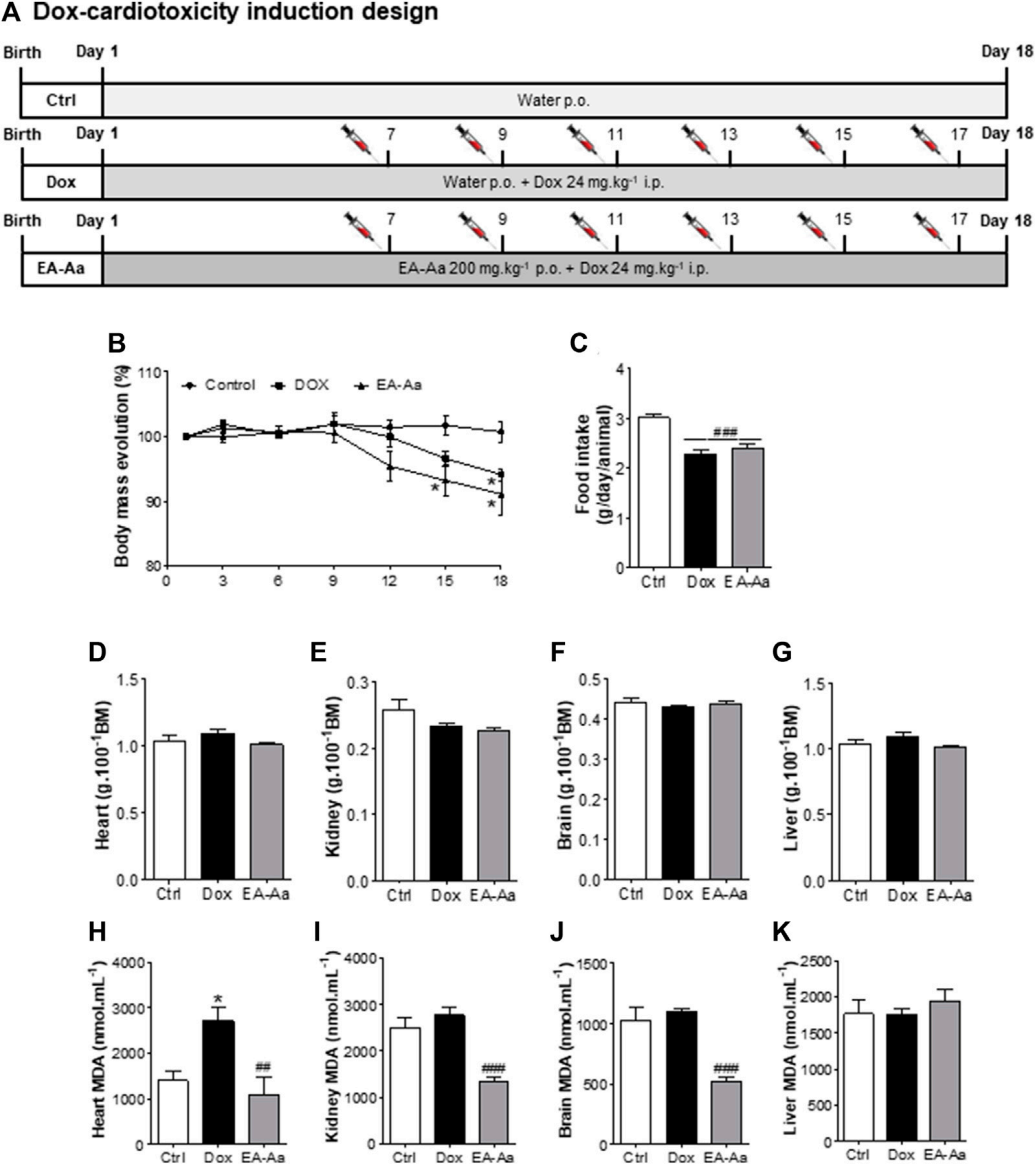
EA‐Aa reduces Dox-induced toxicity in C57Bl/6. Experimental design **(A)**; Bodymass evolution **(B)**; Food intake **(C)**; and weight of heart **(D)**, kidney **(E)**, brain **(F)**, liver **(G)**. MDA levels were determined in the heart **(H)**, kidney **(I)**, brain **(J)**, and liver **(K)**. Ctrl group—control mice; Dox group—Doxinduced mice with 24 mg kg–1; EA‐Aa—Dox‐induced mice treated with EA‐Aa 200 mg kg–1. * vs. Ctrl; # vs. Dox; *p < 0.05; ##*p* < 0.01; ###*p* < 0.001.

Animals treated with EA-Aa 2000 mg kg^−1^ and 5000 mg kg^−1^ did not present any physiological signs of toxicity, such as significant body weight reduction, physical or behavior changes, or mortality (Figure 6). Only a slight increase in liver weight of 26% in comparison to the control, in the higher EA-Aa concentration tested, as presented in Figure 6H, was observed. Hematological changes were observed in the group treated with the higher dose of EA-Aa, with a slight increase in white blood cells and fractions (Table 1). Dox-treated C57Bl/6 mice (Figure 7A) showed weight loss and lowered caloric intake values, compared with the control group, conditions that were not reverted by EA-Aa (Figures 7B, C). The weight of several organs (liver, heart, kidney, and central nervous system) from the same animals did not change in response to Dox or EA-Aa treatment (Figures 7D–G). In turn, MDA levels in animals submitted to the chemotherapeutic agent showed an increase of 94% in the heart, which was completely restored after treatment with EA-Aa, with values 23% lower than the control (Figure 7H). Such protection was also observed in the kidney and brain, with a reduction of 46% and 49% in MDA below baseline levels (Figures 7I, J).

**TABLE 1:** C57Bl/6 hematological parameters of mice treated with single doses of EA-Aa.

	Control	EA-Aa 2000 mg.kg^−1^	EA-Aa 5000 mg.kg^−1^
WBC (10^3^ µL^−1^)	2.1 ± 0.3	3.1 ± 0.3*	3.8 ± 0.3**
RBC (10^6^ µL^−1^)	10.3 ± 0.4	9.5 ± 0.1	8.0 ± 0.6**
HGB (g.dL^−1^)	14.2 ± 0.5	13.5 ± 0.1	11.4 ± 0.7**
HCT (%)	56.5 ± 2.5	52.8 ± 0.6	45.5 ± 2.2**
MCV (fL)	55.0 ± 0.5	55.6 ± 0.6	57.6 ± 2.1
MCH (pg)	13.9 ± 0.1	14.2 ± 0.1	14.3 ± 0.3
MCHC (g.dL^−1^)	25.2 ± 0.3	25.7 ± 0.2	25.0 ± 0.3
PLT (10^3^ µL^−1^)	367.6 ± 238	408.8 ± 168.7	493.8 ± 202.4
RDW-SD (%)	26.5 ± 0.6	26.9 ± 0.4	28.7 ± 1.7
RDW-CV (%)	19.8 ± 0.4	19 ± 0.2	17.4 ± 0.7**
NEUTROPHIL (10^3^ µL^−1^)	0.1 ± 0.0	0.1 ± 0.0	0.0 ± 0.0
LYMPHOCYTE (10^3^ µL^−1^)	1.9 ± 0.2	3.0 ± 0.3*	3.5 ± 0.2**
MONOCYTE (10^3^ µL^−1^)	0.0 ± 0.0	0.0 ± 0.0	0.2 ± 0.1
EOSINOPHYL (10^3^ µL^−1^)	0.0 ± 0.0	0.0 ± 0.0	0.0 ± 0.0
BASOPHIL (10^3^ µL^−1^)	0.0 ± 0.0	0.0 ± 0.0	0.0 ± 0.0

WBC, white blood cells; RBC, red blood cells; HGB, hemoglobin; HCT, hematocrit; MCV, mean corpuscular volume; MCH, mean corpuscular hemoglobin; MCHC, mean corpuscular hemoglobin concentration; PLT, platelet; RDW, red cell distribution width. EA-Aa = Aqueous extract of *A. aculeata* leaves. Data are expressed as mean ± SEM. * vs. Ctrl, *p < 0.05; **p < 0.01; ***p < 0.001.

## 4 Discussion

In this study, we showed that the aqueous extract of *Acrocomia aculeata* leaves decreased the viability of cancer cells, reducing mitochondrial potential and inducing cell death by apoptosis, late apoptosis, and necrosis. Besides the anticancer effect of EA-Aa by itself, it is interesting to highlight that EA-Aa potentiates Dox cytotoxic effect in co-treatment in cancer cells and protects cardiotoxicity against Dox-induced oxidative stress *in vivo.* Dox is one of the best-described anthracyclines used in chemotherapy due to its wide therapeutic efficacy (Wallace et al., 2020), although its side effects are also present, here we highlight cardiotoxicity, the most well-known (Monsuez et al., 2010). Besides the increased levels of proapoptotic and proinflammatory factors and autophagy markers, Dox-induced mechanisms lead to increased ROS/RNS levels (Yarmohammadi et al., 2021) and consequently oxidative stress. Intending to identify new therapeutic alternatives that could prevent chemotherapy side effects connected to oxidative stress, medicinal plants with antioxidant properties have become a promising strategy (Casagrande et al., 2014; Lopes et al., 2016; Santos et al., 2016; dos Santos et al., 2018; Rodrigues et al., 2019; Figueiredo de Santana Aquino et al., 2020).

Accordingly to Negrette-Guzmán, cancer cells generally produce higher ROS levels than normal cells, a feature that stimulates the tumor in its progression and chemoresistance, involving the upregulation of hypoxia-inducible factor-1 alpha (HIF-1α) and nuclear factor-kappa B (NF-κB) (Negrette-Guzmán, 2019). Chemoresistance also involves the regulation of redox-sensitive transcription factors, such as NRF2, which remains in the cytoplasm when linked to the complex Kelch-like ECH-associated protein 1 (Keap 1) and its dissociation and migration to the nucleus requires the activation of some ROS-mediated kinases as ERK, JNK, and p38 or some antioxidants. NRF2 can transcriptionally activate some antioxidant proteins in the nucleus and can upregulate nuclear respiratory factor 1 (NRF1), which increases mitochondrial function (Negrette-Guzmán, 2019). As we already proved in a recent study (Monteiro-Alfredo et al., 2020), EA-Aa increases p-NRF2, p-ERK, and catalase in Cos-7 cells (kidney fibroblasts), and this suggests that this pathway may be at least in part involved in the antioxidant protection of H9c2 cardiomyoblasts promoted by EA-Aa. The observed potentiation of Dox-cytotoxic effects and the induction of mitochondrial dysfunction in erythroleukemia cells (K562 cells) may be related to the antioxidant and NRF2-activating effects of EA-Aa, already described for other sources of phenolic compounds (Han et al., 2008; Peng et al., 2020; Sharma et al., 2020; Kaur et al., 2021; Singh et al., 2022). As proposed in the study of Ojha, the mentioned effects may be related both to the stabilization of the radicals in the electron transport chain (as we show with the JC-1 assay) and to the increase of stress-related protein levels and the NRF2 function, presented by cardiomyoblasts, H9c2 cell (Ojha et al., 2016). Additionally, if the higher levels of catalase help to stabilize the radicals generated by cancer cells, this effect may be probably related to the greater mitochondrial dysfunction in K562 cells, caused by the co-treatment between Dox + EA-Aa, especially at the higher concentrations tested.

When K562 and MCF-7 cells were incubated with EA-Aa and the lower concentration of Dox, the cytotoxic effect was bigger than in cells treated with Dox alone and was similar to the cells treated with the higher dose of Dox. These data show the potentiation of Dox effects by EA-Aa (which occurred mostly through addition) even in the minor concentrations tested, leading to a better chemotherapeutic effect of Dox and significantly lower side effects (Turrini et al., 2014; Mokhtari et al., 2017). Regarding the type of death, data from AV/PI double staining, measured by flow cytometry, supports the cell viability assay, showing that the Dox effect occurs mainly through necrosis in these cells, which may be associated with the mechanism of action of Dox on Topoisomerase II and DNA intercalation, as already described (Shin et al., 2015). Instead, EA-Aa induced mitochondrial dysfunction and increased apoptosis, so we must consider EA-Aa as a probable stabilizer of the redox condition.

Between the phytochemical compounds present in medicinal plants, phenolic compounds and flavonoids have shown, in addition to a relevant cytotoxic effect (de Carvalho et al., 2020), a potential in stabilizing the redox condition present in this type of cells. In a previous study (Monteiro-Alfredo et al., 2020), we elucidated the different compounds presented in *A. aculeata* leaves, such as the phenolic compounds—gallic, caffeic, vanillic, and ferulic acids, and the flavonoids rutin and quercetin. Besides being responsible for the proven antioxidant effect of EA-Aa, this composition may protect from Dox-induced oxidative hemolysis, MDA formation, and Dox-induced toxicity in H9c2 cells. Indeed, the combination of quercetin and Dox was already shown by Mahbub et al. to activate the mitochondrial apoptotic pathway through caspases 3 and 9 activations (Mahbub et al., 2015).

Dox was chosen as an oxidative stress inducer for being a source of peroxynitrite (ONOO^−^), an oxidant agent formed from other two radicals, nitric oxide and superoxide (Ferdinandy, 2006), which is a known inducer of oxidative stress-related mechanisms, such as DNA strand breaking, induction of lipid peroxidation, and inhibition of the respiratory chain (Lebrecht et al., 2007). Considering this, peroxynitrite produced by Dox is highly related to the development of cardiomyopathy, and its inhibition or reduction may be an alternative for a therapeutic combination. Dox also increased MDA levels in cells (Lebrecht et al., 2007), and treatment with EA-Aa reduced this oxidative stress biomarker level by 30% in RBC, which was followed by a 50% reduction of the oxidative hemolysis assay. This protection probably occurs due to the secondary metabolites (ferulic, caffeic, and vanillic acids—(Han et al., 2008; Peng et al., 2020; Kaur et al., 2021) extracted from *A.* aculeata leaves, as mentioned before. One of the aromatic rings in the flavonoid structure has a hydroxyl configuration that donates hydrogen and electrons to molecules such as peroxyl, peroxynitrite, and hydroxyl, stabilizing them, besides the ion-chelating property of quercetin (Kumar and Pandey, 2013).

Flavonoids are relevant to Dox-induced chronic cardiotoxicity (Shabalala et al., 2017) because they prevent both its cytotoxicity and decrease the anticancer effect (Korga et al., 2017). To evaluate the potential of EA-Aa against the most noted side effects of Dox chemotherapy, we performed an *in vivo* assay. First, we proved the non-toxicity of EA-Aa in C57Bl/6 mice, and as we expected, the results showed no relevant markers of acute toxicity for 2000 mg kg^−1^, proving the safety of consumption. Thus, we next used a ten times lower dose of EA-Aa in the animals for the Dox-induced cardiotoxicity test. The cardiotoxicity of Dox occurs when administered in cumulative doses (Mitry and Edwards, 2015), and after the treatment, the EA-Aa group showed a complete reversion of MDA levels concerning the Dox group, showing the cardioprotective effect of the extract and the reduction of MDA in the kidney and brain below the baseline levels.

## 5 Conclusion

Our results demonstrate that in cancer cells, EA-Aa shows a cytotoxic effect and potentiates Dox cytotoxic effect in co-treatment. In addition to not showing signs of toxicity in non-cancer cells, EA-Aa revealed an attenuating effect on Dox-induced oxidative stress in erythrocytes and cardiomyoblast, which is probably associated with decreased cardiotoxicity in C57Bl/6 mice. Together, these data support additional studies to develop a pharmacological adjuvant based on EA-Aa or its chemical constituents.

## Data Availability

The original contributions presented in the study are included in the article/Supplementary Material, further inquiries can be directed to the corresponding author.
